# Barriers to accessing highly active antiretroviral therapy by HIV-positive women attending an antenatal clinic in a regional hospital in western Uganda

**DOI:** 10.1186/1758-2652-13-37

**Published:** 2010-09-23

**Authors:** Putu Duff, Walter Kipp, T Cameron Wild, Tom Rubaale, Joa Okech-Ojony

**Affiliations:** 1 School of Public Health, University of Alberta, Canada; 2 Community ARV-Project, Fort Portal, Uganda; 3 Kabarole District Health Office, Fort Portal, Uganda

## Abstract

**Background:**

The aim of this study was to describe barriers to accessing and accepting highly active antiretroviral therapy (HAART) by HIV-positive mothers in the Ugandan Kabarole District's Programme for the Prevention of Mother to Child Transmission-Plus (PMTCT-Plus).

**Methods:**

Our study was a qualitative descriptive exploratory study using thematic analysis. Individual in-depth interviews (n = 45) were conducted with randomly selected HIV-positive mothers who attended this programme, and who: (a) never enrolled in HAART (n = 17); (b) enrolled but did not come back to receive HAART (n = 2); (c) defaulted/interrupted HAART (n = 14); and (d) are currently adhering to HAART (n = 12). A focus group was also conducted to verify the results from the interviews.

**Results:**

Results indicated that economic concerns, particularly transport costs from residences to the clinics, represented the greatest barrier to accessing treatment. In addition, HIV-related stigma and non-disclosure of HIV status to clients' sexual partners, long waiting times at the clinic and suboptimal provider-patient interactions at the hospital emerged as significant barriers.

**Conclusions:**

These barriers to antiretroviral treatment of pregnant and post-natal women need to be addressed in order to improve HAART uptake and adherence for this group of the population. This would improve their survival and, at the same time, drastically reduce HIV transmission from mother to child.

## Background

Sub-Saharan Africa has been devastated by the HIV/AIDS epidemic, having more infections and deaths than any other region globally [1]. Uganda is among those countries in the sub-Saharan region that is hit hard by HIV/AIDS: the latest national HIV prevalence is estimated to be 5.4% among adults, and higher among women (7.5%) than among men (5.0%) [2]. Since there are currently an estimated 940,000 people living with HIV in Uganda, there is potentially a high demand for highly active antiretroviral therapy (HAART) in this population: Hladik *et al *estimate that 111,100 HIV patients will require HAART in Uganda by the end of 2010 [3].

Unfortunately, only a small portion of HIV patients actually receive HAART. This is in spite of the strong efforts of the Ugandan Government to scale up HAART programmes, including an extensive information and education campaign. Hladik *et al *also stated that universal access to HAART may not be achievable in Uganda at all [3]. Efforts to increase access to HAART are therefore crucially important and it is paramount to assess all factors currently restricting access to HAART. With this as a first step, HAART programmes can be modified to facilitate easier access and attempt to provide universal HAART as a fundamental service to all HIV/AIDS populations.

Previous studies in resource-poor countries have identified a number of factors that hinder access to HAART for both men and women in sub-Saharan Africa. Factors identified and ranked high as serious obstacles to accessing HAART were transportation costs in Uganda and Tanzania [4-6]. In Benin, South Africa and Malawi, restricted access was due to complicated dosing in HAART regimes, language barriers for patients communicating with staff, and economic reasons [7-9]. Similar findings were reported from Zambia, where high costs of treatment (e.g. laboratory tests), adverse reactions to HAART, an overcrowded health care system, overworked clinic staff and language problems were reported to hinder the uptake of HAART [10]. In a comprehensive review of barriers to HAART access, the most cited barriers were lack of information about HAART, including knowledge about treatment procedures, and a lack of coordination between HAART services and other health care services [11].

Regarding access to HAART for women and specifically for pregnant or post-natal women, much less information is published. A multi-centre study with 33,164 individuals from 13 countries, including participants from sub-Saharan Africa, concluded that enrolment in HAART was higher for all women compared with men, and that enrolment had increased in the past years to 68% [12]. In contrast to this review, several studies reported that pregnant and post-natal women had less access to HAART or did not want to utilize it. One study from Zambia included breastfeeding women and found that (as in other studies) stigma, adverse reactions to HAART and lack of food were barriers to accessing HAART. Other factors not previously described in the literature were the presence of mental disorder (e.g., depression), hopelessness and the potentially damaging effect of HAART on personal relationships, particularly between wife and husband [13]. A study from Malawi reported that pregnant women (among other groups, such as TB patients and children) accessed HAART less often than other HIV patients [9]. In Wakiso District, Uganda, prevention of mother to child transmission (PMTCT) programme coverage for pregnant and/or post-natal women was reported to be as low as 28% [14].

In order to address gaps in knowledge for understanding barriers to HAART among pregnant and breastfeeding women, we conducted our study in Kabarole District, western Uganda, with a sample of pregnant and post-natal women attending the Prevention of Mother to Child Transmission-Plus (PMTCT-Plus) programme run by the regional government hospital in Fort Portal. We focused on pregnant and post-natal women because they are especially vulnerable and have generally received less attention than other populations.

The Ugandan PMTCT-Plus programme is an initiative to scale up HAART uptake by pregnant and post-natal women and to reduce mother to child transmission of HIV. It consists of provision of free HIV counselling and testing, single-dose nevirapine and/or combination HAART to eligible HIV-positive mothers and family members, using eligibility criteria according to the national guidelines for HAART [15]. Despite the Ugandan Government's efforts to scale up the PMTCT-Plus programme from 2003 to 2006, 22,000 new paediatric HIV infections due to mother to child transmission were recorded in recent years [16].

It is crucial to the success of the Kabarole PMTCT-Plus programme that locally perceived barriers to accessing and accepting HAART are comprehensively described, understood and addressed. This study explores PMTCT-Plus clients' barriers to enrolling in the programme, as well as obstacles faced by those who have enrolled and want to continue with HAART. The specific objectives of our study were to: (1) explore and describe barriers to accessing free HAART among women in Kabarole's PMTCT-Plus programme at a regional hospital; (2) analyze women's positive and negative HAART experiences within the context of the delivery of the existing PMTCT-Plus programme; and (3) use the study findings to make recommendations to improve the uptake of HAART by women attending this programme.

## Methods

This was an exploratory, descriptive qualitative study using thematic analysis. Data were collected through in-depth interviews and one focus-group discussion. We chose this approach because using a qualitative methodology could elicit extensive discussions that revealed women's perceived barriers to HAART in a way that may not have been obtained from solely quantitative methodologies.

### Study setting

Kabarole's PMTCT-Plus programme was first launched in 2003 in five hospitals. These hospitals are attended by 13,000 women, 8% of whom are HIV infected. Kabarole District's PMTCT-Plus programme at the regional hospital experiences a low uptake of HAART among eligible PMTCT-Plus clients, with approximately one-quarter of the clients on HAART lost to follow up in 2006 (personal communications, J. Okech, Kabarole District Health Officer, Fort Portal, Uganda). Kabarole District has an estimated population of just under 400,000, approximately 20,000 of whom are pregnant women at any given point in time [17]. The district's population is characterized as low income, with subsistence farming representing the main source of income. Approximately 74% of the population resides in rural areas, far from hospitals and health clinics that are located in the urban centres [18].

### Study sample

Study participants were identified using the PMTCT-Plus client register at the government-run referral clinic for the district, based at the regional hospital. All programme registrants were categorized into four groups: (1) clients who were eligible for HAART but never enrolled in the programme; (2) clients who formally enrolled in HAART but never returned to start HAART; (3) clients who enrolled and defaulted on HAART; and (4) clients taking HAART medication during the time of the interviews without reported interruption. Women were eligible to participate in the study if they had attended antenatal care and received pre-test HIV counselling and were found to be HIV positive, and were 18 years and older. Each registrant was assigned a number. Study participants were chosen via simple random sampling in each group separately (see Table 1).

**Table 1:** Group classification of study participants

Group	Interviews (45)	Focus group discussion (one)	Total
1) Never enrolled for HAART	17	4	21
2) Enrolled but never began HAART	2		2
3) Defaulted HAART	14	4	18
4) Taking HAART	12		12
**Total**	**45**	**8**	**53**

Potential study participants were contacted by a research assistant in a discreet manner, informed about the study, and were given an information letter describing the purpose of the research, confidentiality and the right to refuse to participate. There was a high acceptance rate, with only two women out of 53 declining to be interviewed. Respondents were questioned via in-depth interviews, and another eight randomly selected respondents were included in one focus group discussion (FGD). The FGD was conducted following the interviews and was designed to enhance trustworthiness of the findings by validating results obtained from the interviews.

### Data collection and analysis

In-depth, face-to-face interviews and the FGD were conducted using an interview guide with open-ended questions. The topics were derived from findings on barriers to HAART access in the literature, along with advice provided by health professionals and experts in the field (in Uganda and in Canada). The interview and FGD guides included such topics as: personal views about the PMTCT-Plus programme; HAART knowledge; perceived impact of HAART on patients and family members; social support for patients; experiences of HIV/AIDS stigma; issues related to HIV serostatus disclosure to participants' partners; patient and health care delivery factors; perceived barriers to accessing HAART services (e.g., geographical distance, economic and time constraints); fear of HIV/AIDS stigma; and perceived barriers to adhering to HAART regimen (e.g., forgetfulness, tablets not refilled, tablets out of stock, adverse reactions to tablets). In addition, those participants who were on HAART but who defaulted later from treatment were asked about their experiences taking HAART, including what led to their decisions to discontinue treatment.

Interviews and the FGD were carried out in quiet and discreet locations, often a vacant room in the hospital's outpatient department, where the interview could be carried out privately and without interruption. The interviews were conducted and audio-taped in the local language, Rutooro, and lasted about 80 minutes each. The FGD was approximately 90 minutes in duration. Tapes were transcribed verbatim in Rutooro and then back-translated into English. Spot checks of interview and FGD transcripts and translations were regularly conducted to ensure the completeness of the transcription and the accuracy of the translation. Data collection was carried out from September to December 2006.

Data analysis for both the interviews and the FGD was conducted using principles of thematic analysis [19,20]. This included multiple readings of the transcripts to capture context and meaning, followed by coding and categorization of recurring concepts and ideas. A master list of all categories were assembled and examined for common themes. Categories of codes were then organized into overarching themes. Data verification was done by a second researcher, who also coded all transcripts. Codes were compared and added or removed based on the agreement between analysts. The results of our interviews were also compared with the literature and verified with participants who were contacted again after the results became available.

Following coding, a frequency distribution list was developed, and the number of responses for each category of participants was recorded and tallied. This allowed us to identify the most frequently mentioned barriers and the proportion of participants who identified an issue as a barrier to treatment. Relative frequency of each thematic issue identified during analysis was calculated and expressed as percentages or in such statements as "most of the participants" or "all of the participants". Similar to the in-depth interviews, the FGD transcript was analyzed using the principles of thematic analysis and compared to the interviews. Recurring topics emerging from the FGD were noted and served to validate data obtained from the interviews. The quotations selected were those that best represented the ideas voiced by participants and were also chosen based on the frequency with which they were mentioned.

### Ethical considerations

Ethical and administrative approval of the study methodology was granted by the Health Research Ethics Board (Panel B) at the University of Alberta, Edmonton, Canada, the Uganda Council for Science and Technology, Kampala, Uganda, and the District Health Officer of Kabarole District. Written, informed consent was obtained from each participant prior to the interviews.

## Results

Most (70.6%) of the participants had very little formal education, were married and were rural dwellers living in economically deprived environments (Table 2).

**Table 2:** Demographic characteristics of study participants

	**Group 1** **(n = 17)**	**Group2** **(n = 2)**	**Group 3** **(n = 14)**	**Group 4** **(n = 12)**
**Average age**	30	36	32	35
**Marital status**				
Married (%)	70.6	100	42.9	41.7
Single (%)	5.9	NA	35.8	16.7
Widowed, separated (%)	23.5	NA	21.3	58.4
**Residence**				
Urban	29.4	0	7.1	83.3
Rural	70.6	100	92.9	16.7
**Educational level**				
No formal education	23.5	100	28.6	8.3
Some primary	52.9	NA	57.1	50.1
Complete primary	11.8	NA	NA	33.3
Some secondary	11.8	NA	14.3	8.3

The perceived barriers described by participants fell under the following five broad themes, ranked here in order of the frequency with which they were provided by respondents: economic factors (e.g., lack of transportation); social/environmental factors (e.g., stigma towards HIV/AIDS); health care factors (e.g., long waiting times); HIV/HAART knowledge; and HIV disease progression (e.g., physical constraints to attending clinics regularly as an outpatient). No new themes emerged from the FGD following the individual interviews, and thus, quotations are presented from both data sources. Similarity of the results obtained from interviews and FGD suggested that the themes derived in the analysis were trustworthy.

### Economic constraints

Lack of finances emerged as the greatest barrier to taking HAART among all four groups. Costly transportation fees for monthly check ups was cited as the most prevalent barrier to enrolling in the PMTCT-Plus programme and adhering to HAART, with 42 of 45 (93%) respondents citing this as the major barrier:

[The reason I defaulted was] the transport issue. Sometimes the transport money would not be enough and I would miss coming so I would feel like if I miss one month, then I should completely give up on HAART. (defaulted HAART)

The majority of participants lived in rural areas distant from the PMTCT clinic and had to rely on costly public transportation to meet appointments. Furthermore, subsistence farming was the main source of income for the majority of participants, which means that having cash is rare:

What caused me to default HAART was poverty in our homes. You don't have a goat, you don't have a cow, you don't have a chicken and you don't have a job, then where does one get the money for coming here to collect drugs and then going back home? (defaulted HAART)

Other financial constraints expressed by participants were the cost of food while waiting to see health care providers and the cost of nutritious foods that HAART patients are recommended to eat while taking the medication. Although these factors represented challenges to taking HAART, they were not cited as reasons for not starting or continuing HAART.

In addition, many respondents indicated that they were economically dependent on their husbands, who either provided or controlled the household finances:

I don't earn anything. We are all looking to my husband. Sometimes he sells a bunch or two of matooke and from that little money we can buy salt and any other needs. (defaulted HAART)

The man cannot give me money; he would rather use it to drink. He says that he doesn't have money, even when you are sick. (never enrolled in HAART)

This was found to limit some women's control over treatment-seeking decisions and ability to begin and adhere to HAART. Economic dependence on spouses was a particular barrier among those women who had not disclosed their HIV-positive status to their partners:

If I told my husband I were HIV positive he would stop buying food and drinks and that is why I decided to keep quiet and I used to take my drugs secretly. If I told him he would have stopped all forms of assistance...(defaulted HAART)

### HIV-related stigma

Most patients acknowledged that HIV/AIDS stigma was prevalent within their communities and that people living with HIV/AIDS were discriminated against due to their positive HIV status. One of the 53 women interviewed claimed that she never enrolled in HAART in order to hide her positive HIV status from her community. Despite this, many (58%) respondents claimed that the community's view of HIV-infected persons had no bearing on their decision to begin or continue treatment:

What people think does not hinder me because this life is mine. Let them talk. I'm not the first one to have HIV. (taking HAART)

### Non-disclosure of HIV serostatus

Non-disclosure of a client's HIV-positive status was the second most cited barrier to enrolling in the programme and continuing treatment. Ten of the 53 respondents said they had withheld their HIV status from their sexual partners. The challenge of disclosure was reported to stem from their partners' reluctance to test for HIV:

I did not tell my husband because he refused to go for a check up with me, so I decided to wait to start these drugs because when I tell him he will say I am the one who brought the disease. (never enrolled in HAART)

The women explained that they withheld their status from their partners out of fear of blame, domestic violence, abandonment, divorce and loss of economic support that might ensue:

The reason why I did not tell my husband is that I thought that when he sees me taking the drugs he will say that I am the one who has brought the disease and he will beat me. I was taking the drugs secretly and thereafter said to myself, how long will I keep hiding the drugs, and I decided to leave the drugs. (defaulted HAART)

My child was very young and I knew that if I told [my husband] he would run away from me and leave me suffering with the child. So that is why I fear to tell him. (never enrolled in HAART)

Several participants disclosed that hiding their HIV status made avoiding unprotected sex with their partners a challenge. Despite knowing that practicing safe sex may be important to prevent re-infection and viral resistance to HAART, several participants stated that suggesting safe sex might give rise to suspicion in their spouses. Fear of exposing their status through the suggestion of safe sex was cited as a barrier to beginning and continuing HAART:

He might find out about my HIV status if I want to use a condom. I haven't decided yet since I haven't yet started on treatment. I still fear him. (enrolled but never began HAART)

I realized that I was wasting my time because I was taking HAART and having unprotected sex. It was a waste of time because I am not supposed to be having unprotected sex while on HAART. (defaulted HAART)

### Health care service factors

HAART health care service factors, such as long wait times and negative interactions with the staff, represented barriers to continuing HAART, expressed by Group 3 and 4 participants. Waiting times were reported on average at least four hours, but ranged from one hour to longer than a day:

The reason why I stopped taking my drugs is because I would sometimes come and the services at the clinic wouldn't be good. We would spend the whole day at the clinic and you don't even see the nurse and you even end up not getting the drugs and you go back home empty handed. I waited so long and no one was giving me the drugs. I decided to go home. (defaulted HAART)

Eight of the 24 clients (33% of participants) reported very negative interactions with programme staff, such as rude comments and unacceptable behaviour, such as shouting. Almost all of the remainder cited suboptimal interactions with the staff as a barrier to continuing HAART:

The staff shouts at you in the midst of other patients and they say "Did I give you AIDS?" It is so hurting. (taking HAART)

Staff's favouritism of some patients was cited as a barrier and contributed to long wait times and failure to receive the drugs during the monthly visits. Patients who were not familiar with the clinic staff or were new on treatment were especially neglected:

There is one problem I have noticed. When you come here and you are not known to anybody, you can end up going home without receiving drugs. (defaulted HAART)

### HIV/AIDS and HAART knowledge

Participants who had enrolled for HAART (Group 3 and 4) articulated a level of HIV/AIDS and HAART knowledge sufficient to make an informed decision to continue or terminate their treatment. Counsellors and health providers were identified as the most common source of reliable information:

The doctor taught me again and again and every time we come back for drugs they again told us about the drugs. So we understand. (defaulted HAART)

Respondents who had not enrolled for HAART demonstrated a markedly lower level of HIV/AIDS and HAART knowledge compared with those who had enrolled in the PMTCT-Plus programme. Patients explained that instead of one-on-one counselling, they were provided with pamphlets about HAART, which many didn't read because they were illiterate or were embarrassed to take them home where others may find and read them:

I didn't go back to enrol so I haven't been counseled about HAART yet. So how can I know about HAART? (never enrolled in HAART)

I won't lie. I haven't kept those books for fear that someone will come and read them. They will find out what I am. The information was there but I didn't read it. (never enrolled in HAART)

Many patients referred to radio shows and casual village conversation/gossip as their main sources of information about HIV/AIDS and HAART. This can be problematic, as village conversation, in particular, has proved to be fraught with fallacies. The most common misconception was that HAART makes the patient weak and actually kills those ingesting it:

People say that drugs make you lose energy, and then you die. That's what people say. (never enrolled in HAART)

Now you see that if you are taking drugs you can die there and then. I have to control myself and look after my kids to see that they've grown, work for them and build for them. Now you see my land is not progressing, we want to build. Do you want to kill me there and then? (never enrolled in HAART)

### Patient's physical health and HIV disease progression

More than half of the patients who had not enrolled in HAART claimed that they were deferring treatment until their health deteriorated significantly. Many participants (60%) perceived that the expected time to be receiving HAART was when they became bedridden:

How can I start on the drugs and yet I am not yet bedridden and I have not felt anything and not seen any symptoms..." (never enrolled in HAART)

A number of respondents explained weighing one's physical state against the availability of finances when making the decision to start HAART:

It depends on how I feel in my life. What can force me to start HAART is money because when I have money and feel that I'm not OK, then I can start. (never enrolled in HAART)

## Discussion

This study described the cultural context and locally perceived barriers to accessing HAART among HIV-positive women attending the PMTCT-Plus programme at the main regional hospital in Kabarole District. As the perception of pregnant and post-natal women regarding access to HAART has not been well documented previously, we believe that the information in our article contributes to improving access for treatment of this vulnerable group. Because pregnant and post-natal women who are successful on HAART have little risk of transmitting their HIV infection to their babies, this programme has huge relevance for the wellbeing of the women being treated, their infants and their entire families. In contrast to other studies from sub-Saharan Africa, this study included participants who were lost to follow up by the health care providers in the Kabarole PMTCT-Plus programme, and therefore we were able to capture barriers to HAART among an understudied group.

Consistent with other studies of groups other than pregnant women, financial constraints, such as high transportation costs represented the major barrier to starting and adhering to HAART [5,21,22]. High transportation costs were found to be the most formidable barrier to treatment by the authors of these studies and also by us. Therefore, proximity to the clinic emerged as a strong determinant of access, with the majority of participants not taking HAART residing in rural areas, too distant from the clinics providing HAART. This is a problem for the majority of Kabarole's population, including pregnant women who reside in rural areas, where only 26.1% of them are within 5 km walking distance of health care facilities [23]. Efforts to reduce or eliminate this barrier would likely substantially increase HAART acceptance and adherence. As it is not feasible to increase access to HAART by building new clinic facilities in poor countries, such as Uganda, or by providing universal access through formally trained health care workers alone, as suggested by Barnighausen, the only option is to bring HAART services closer to the population through community resources and community premises [24].

In three Ugandan studies, it has been shown that this is possible. Two HAART studies come from eastern Uganda (one from Jinja, run by the Medical Research Council UK and The Ugandan Support Organization [22], and one from Tororo, run by the Centres for Disease Control and Prevention, USA), where home-based HAART services were offered and excellent treatment outcomes were achieved [25]. Treatment success was measured by the HIV-1 RNA viral load (VL) in patients: in both studies, more than 90% of patients had suppressed VL. A third study was run in Mbuya, a suburb of Kampala, by the Anglican Church of Uganda, where lay persons were involved in community-based HAART services, providing an outreach treatment, monitoring and adherence support programme [26]: treatment success as measured by VL was similar to that found in the other two studies.

These three examples of programmes show that community/home care-based approaches to HAART are feasible and bring HAART services closer to the population. One other potentially useful option for expanding HAART services in Uganda, recently suggested by Mbonye *et al*, was to use private midwives as the delivery point for HAART services to pregnant and post-natal women [27].

Social factors were identified as the second most cited and important barrier to HAART access. The study participants revealed non-disclosure of their HIV status to their partners, and directly attributed this to HIV-related stigma; most asserted that this was a substantial barrier to treatment. Non-disclosure to partners as an impediment to treatment has been recognized in other African studies, particularly pertaining to adherence [4,28,29]. Nachega *et al and *Olley *et al *found the rate of partner non-disclosure to be 22% and 38%, respectively, in their South African studies [28,29]. Similar to findings from other studies, non-disclosure by our participants arose from fear of blame, violence, abandonment, divorce and loss of economic support [30-32]. Our data demonstrated this fear to be legitimate: several respondents described that such incidences occurred following disclosure to their partners.

Health care delivery issues were the third most frequently cited barrier to access HAART. This reflects the underlying issue of human resource constraints, pervasive in busy HAART clinics in sub-Saharan Africa. This issue has been identified as an important health system limitation to scaling up HAART [33,34]. An inadequate supply of health care personnel to meet rising demands leads to long waiting times, and overburdened and overworked health care workers who take their frustrations out on their patients. Studies have shown that women, especially, are more likely to participate in programmes if health care workers have positive attitudes [35,36].

Respondents' knowledge about HIV/AIDS and HAART was generally high. There were no significant discrepancies in HAART knowledge among those taking HAART and defaulters. These results are contrary to conventional views, which often hold that knowledge of treatment effectiveness and procedures to be followed is associated with better HAART uptake or adherence. Our data suggest that financial constraints may be so overwhelming that they prevent access to HAART, regardless of patients' HAART knowledge and intentions to take the drugs. Participants who had not enrolled for HAART did demonstrate a suboptimal knowledge of HIV/AIDS and HAART, which acted as a barrier for some. Several respondents exhibited deficits in HAART knowledge in that they felt HAART was required only after clinical symptoms due to HIV had developed. Early start of HAART has been found to be associated with better survival [37].

There were several limitations associated with this study. First, as this was a qualitative study, the results are not intended to be generalized to the population at large. Second, social desirability bias cannot be excluded, as the study topic dealt with sensitive issues. This could potentially alter participants' responses, causing them to provide answers that conform to socially accepted norms. To reduce this bias, highly trained interviewers familiar with qualitative interview techniques were used. Third, the study was carried out in 2006, and how pregnant women access HAART now may be different to what we described. Generally, we think that these limitations do not significantly compromise the validity of our study.

We provide the following recommendations to the Kabarole Health Department to mitigate the four most important barriers to HAART access, as found in this study:

### Develop and expand community-based HAART services

Economic costs associated with HAART, such as transport to the nearest HAART clinic, food requirements during long waiting times, and loss of time, can only be realistically reduced by moving HAART services closer to the people in need. It also needs a community-based approach, where routine tasks, such as monitoring HAART and supporting adherence, is shifted to lay persons in the community. Barnighausen reported that formally trained health care workers will not be able to provide universal access to HAART in the next decade [24]. A few successful models already exist in Uganda, as we have mentioned.

### Strengthen a programmatic approach to HIV-related stigma

It is possible to promote HIV/HAART counselling to both male and female partners in joint counselling sessions. Health care workers and/or counsellors could be better prepared to provide this partner-based service more effectively, which would address HIV-related stigma at the most personal level. Programme guidelines would have to be established. It would also require some resources, motivation and persistence from staff to make it happen. In general, men have been very willing to participate in reproductive health/HIV/AIDS education.

### Improve HAART services management

Overburdened HAART clinics and burned-out HAART workers are understandable as the demand for HAART far outweighs what the infrastructure can provide. However, better management of HAART clinics could ease the situation, e.g., for those patients who said that they had to stay overnight near the hospital as they were asked to come back the next morning, or for those who reported having to wait for a long time because their files were "lost". Negative staff attitudes and behaviour expectations should be monitored by the staff supervisors and embedded in a "no tolerance policy for patient abuse".

### Educate widely for early HAART initiation

The misconception of some study participants that HAART is required only after clinical signs of HIV infection have begun is dangerous. It can lead to a delay in treatment with less favourable survival outcomes. To correct this misconception, a simple education component could be inserted into all of the existing HIV/AIDS treatment and counselling services, as this applies to all HIV-positive persons, not only to women.

While these four recommendations are key to the success of all HIV/AIDS-related programmes, not only for HAART, they reinforce the urgency that should exist in addressing this specific target population attending PMTCT-Plus programmes. With all the gender issues known to be important for women with HIV/AIDS, treating these women effectively would improve their own individual health, the health of their children and the health of other family members. The final three recommendations could be implemented in the existing service delivery model of clinic-based HAART, and need only a stronger quality-improvement, client-focused approach. A community-based district HAART programme, with government support for improved HAART access, requires a major shift in service delivery for rural communities, for which successful models already exist.

## Competing interests

The authors declare that they have no competing interests.

## Authors' contributions

PD was involved in study design, field work execution, data analysis, interpretation and writing the first draft of the article. WK helped with study design, data analysis and interpretation and wrote the final version of the manuscript. CW was involved in the study design, data analysis, interpretation and gave input into the final version of the article. TR provided input into the study design, supervised the field work and commented on the study results and their interpretation as well as the first draft of the article. JO-O supervised the field work and provided comments on the study results and their relevance to the health care services as well as commented on the first draft of the article. All authors have read and approved the final manuscript.
